# Post-Heading Heat Stress in Rice of South China during 1981-2010

**DOI:** 10.1371/journal.pone.0130642

**Published:** 2015-06-25

**Authors:** Peihua Shi, Liang Tang, Lihuan Wang, Ting Sun, Leilei Liu, Weixing Cao, Yan Zhu

**Affiliations:** National Engineering and Technology Center for Information Agriculture, Jiangsu Key Laboratory for Information Agriculture, Jiangsu Collaborative Innovation Center for Modern Crop Production, Nanjing Agricultural University, Nanjing, Jiangsu, 210095, P. R. China; Tennessee State University, UNITED STATES

## Abstract

Frequent extreme heat events are the serious threat to rice production, but the historical trend of heat stress associated with phenology shift and its impact on rice yield over a long period are poorly known. Based on the analysis of observed climate and phenology data from 228 stations in South China during 1981-2010, the spatio-temporal variation of post-heading heat stress was investigated among two single-season rice sub-regions in the northern Middle and Lower Reaches of Yangtze River (S-NMLYtz) and Southwest Plateau (S-SWP), and two double-season early rice sub-regions in the southern Middle and Lower Reaches of Yangtze River (DE-SMLYtz) and Southern China (DE-SC). Post-heading heat stress was more severe in DE-SMLYtz, west S-NMLYtz and east S-SWP than elsewhere, because of rice exposure to the hot season during post-heading stage. The spatial variation of post-heading heat stress was greater in single-season rice region than in double-season early rice region due to the greater spatial variation of heading and maturity dates. Post-heading heat stress increased from 1981 to 2010 in most areas, with significant increases in the east of double-season early rice region and west S-SWP. Phenology shift during 1981-2010 mitigated the increasing trends of heat stress in most areas, but not in west S-SWP. Post-heading heat stress played a dominated role in the reduction of rice yield in South China. Grain yield was more sensitive to post-heading heat stress in double-season early rice region than that in single-season rice region. Rice yield decreased by 1.5%, 6.2%, 9.7% and 4.6% in S-NMLYtz, S-SWP, DE-SMLYtz and DE-SC, respectively, because of post-heading heat stress during 1981-2010, although there were some uncertainties. Given the current level and potential increase of post-heading heat stress in South China, the specific adaptation or mitigation strategies are necessary for different sub-regions to stabilize rice production under heat stress.

## Introduction

With the intensification of climate change, short episodes of extreme high temperature events become more and more frequent around the world [1–3]. High temperature above the tolerance threshold of crop growth can cause heat stress, which has greatly negative impacts on grain yield and quality [4–6]. Nevertheless, heat stress in general has not been seriously addressed when estimating the effects of climate warming on agricultural production [7–9]. Recent studies indicated that frequent heat stress events with a warming climate will pose great risks on crop yield stability [10–12].

Rice is a staple food for more than half of the world’s population. As the largest rice producer in the world, China contributes about 28% of the world rice production with 18.5% of the planting area [13]. The growing season for rice in most area of China experiences the hot summer in a year. Heat stress, particularly during the crop reproductive period, could result in dramatic yield reductions [5, 14]. Previous studies indicated that the optimal temperature of grain filling and seed setting in rice ranged from 22°C to 28°C [15, 16]. Short episodes of heat stress during flowering period could induce the failure of pollination caused by the poor theca dehiscence, and result in significant declines of seed number and grain yield [17, 18]. Heat stress during grain filling stage could reduce assimilate supply from shoot to grain, shorten grain filling duration, and eventually decrease grain weight and yield [15, 19]. Heat stress occurs during crop growth period concomitantly with a complex spatio-temporal variation. Spatially, heat stress varies with climate condition, or land suitability for crop production [12]. Temporally, the exposure of crop to heat stress is affected by the sowing time and the developmental rate [12, 20]. Hence, analyzing the spatio-temporal variation of post-heading heat stress and its impact on rice grain yield is important to ensure food security.

In recent decades, ongoing climate change has increased not only average temperature, but also temperature variation [21–23], which might result in more extreme temperature events, like heat stress, across the main rice planting region in China. A wide-spread severe heat event with high temperature of 40°C for 16 consecutive days during grain filling stage was recorded in most areas of the middle and lower reaches of Yangtze River in 2003, which resulted in rice yield losses of 30% [24]. A similar heat stress event occurred during the flowering or grain-filling stage of single-season rice in 2013, which caused a great reduction of spikelet fertility in many counties of South China [25].

Normally, the seriousness of heat stress occurred in crops is associated with temperature, humidity, wind speed and water condition. Among these factors, temperature plays the most important role on rice production under climate change. Many studies estimated the effects of average temperature on rice production [26–28]. However, the effects of heat stress on rice production were not well investigated. Climate warming with increasing temperature variation might cause more heat events across China, particularly in some general wamer areas. Hence, estimating the effects of heat stress on rice production among different eco-regions is of great importance to provide the adaptation or mitigation strategies. In addition, rice phenology varies with different eco-regions and different climatic conditions across China. Previous studies on the analysis of heat stress generally used a fixed phenological date among different eco-sites or growth seasons [29, 30], which probably resulted in the underestimation or overestimation of spatial and temporal variations. Therefore, considering phenology shift is crucial to evaluate the impacts of heat stress on rice production among different eco-regions.

The objectives in this study are: (1) to investigate the spatio-temporal characteristics of post-heading heat stress by calculating heat stress indices from 228 stations during 1981–2010 in the major rice planting regions of South China; (2) to analyze the effects of phenology shift on the trends of post-heading heat stress in rice among different sub-regions; (3) to determine the effect of post-heading heat stress on rice grain yield in South China during 1981–2010.

## Materials and Methods

### Sites and data selection

**Fig 1:**
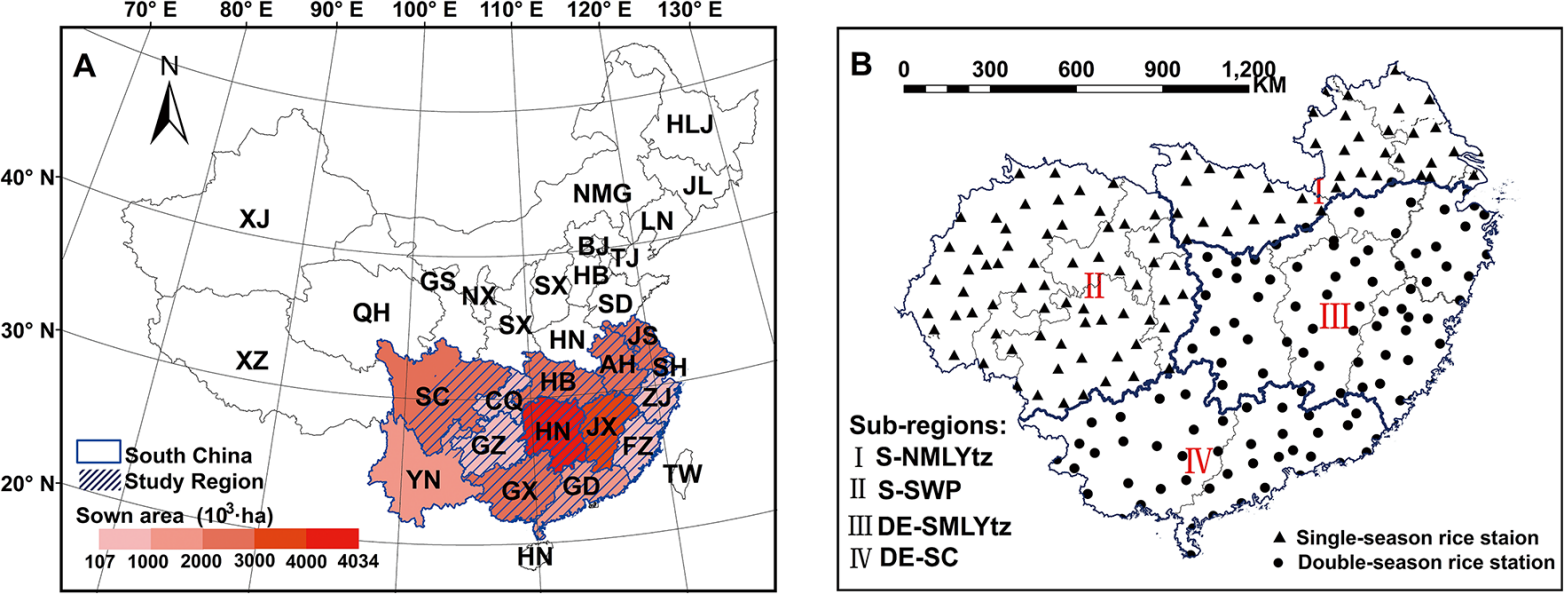
Study region (A) and location of weather stations (B). The sown areas of rice among different provinces or municipalities in Fig 1A are the average values from 2008 to 2012. Triangles and dots in Fig 1B indicate the weather stations in single-season rice region and double-season rice region, respectively. Abbreviations of province or municipality are as follows: JS, Jiangsu; SH, Shanghai; AH, Anhui; HB, Hubei; CQ, Chongqing; SC, Sichuan; ZJ, Zhejiang; JX, Jiangxi; HN, Hunan; GZ, Guizhou; YN, Yunnan; FZ, Fuzhou; GD, Guangdong; GX, Guangxi. Abbreviations of the four sub-regions are as follows: S-NMLYtz, single-season rice sub-region in the northern Middle and Lower Reaches of Yangtze River; S-SWP, single-season rice sub-region in Southwest Plateau; DE-SMLYtz, double-season early rice sub-region in the southern Middle and Lower Reaches of Yangtze River; DE-SC, double-season early rice sub-region in Southern China.

The study areas included 14 major rice production provinces or municipalities in South China as proposed by the Ministry of Agriculture in China in 2009 (http://www.agri.gov.cn). We excluded the regions where rice planting was impossible (northwestern Sichuan with accumulated temperature ≥10°C less than 2000°C·d) or where no heat stress occurred (most areas of Yunnan where the historical maximum temperature as 33.5°C) (Fig 1A). Rice cultivation in this area accounts for about 81% of planting area and provides 84% of rice production in China. The sown area of rice in recent 5 years (2008–2012) is 1,727,000 hectares (ha) on average in the whole study region, ranging from 107,000 ha in Shanghai (SH) to 4034,000 ha in Hunan (HN) (National Bureau of Statistics of China). According to the ecological regionalization of rice in China, four rice planting sub-regions were classified across the whole study region, including two single-season rice planting regions, the northern Middle and Lower Reaches of Yangtze River sub-region (S-NMLYtz) and the Southwest Plateau sub-region (S-SWP), and two double-season early rice planting regions, the southern Middle and Lower Reaches of Yangtze River sub-region (DE-SMLYtz) and the Southern China sub-region (DE-SC) (Fig 1B). In the double-season rice planting regions, only heat stress during the early rice season was analyzed in this study, because normally no heat stress after heading was observed during the late rice season [30].

228 weather stations including 105 single-season rice planting sites and 123 double-season early rice planting sites were selected to calculate heat stress indices (Fig 1B). Historical daily maximum temperature data at each weather station in the study region from 1981 to 2010 were obtained from the Chinese Meteorological Administration (CMA).

**Fig 2:**
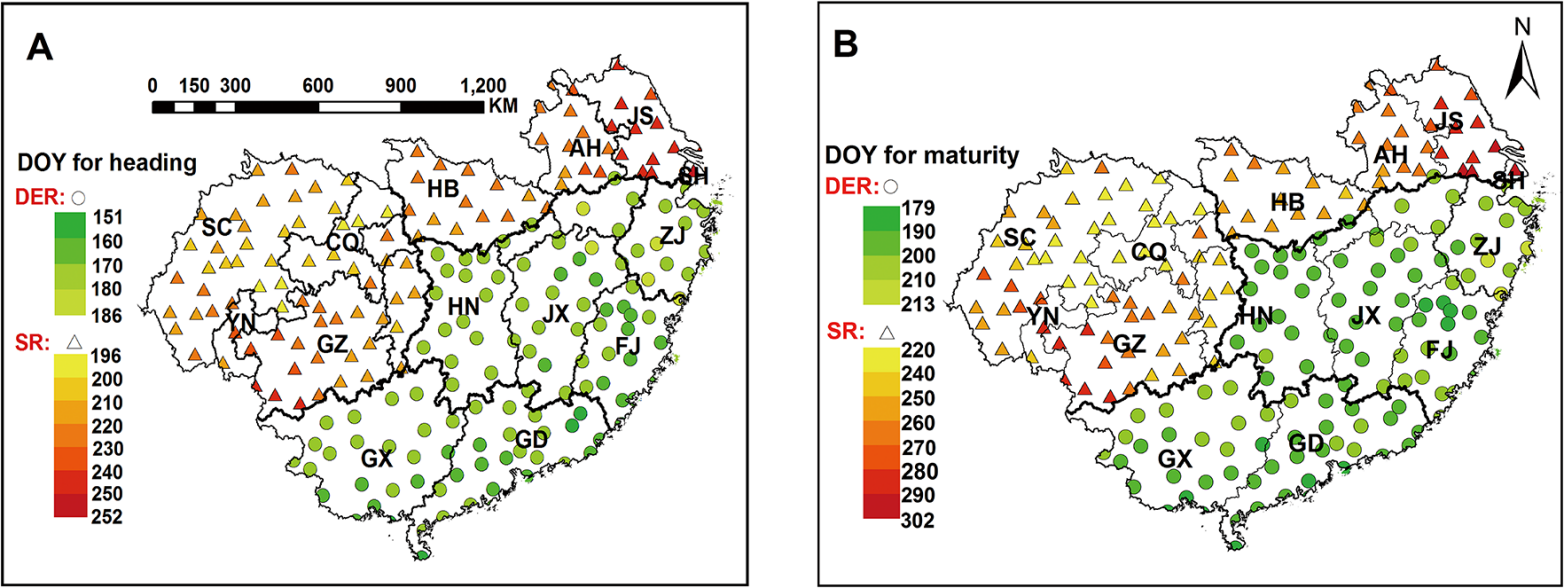
Average DOY for heading and maturity dates from 1981 to 2010 at each station. DOY, day of year; DER, double-season early rice; SR, single-season rice.

**Fig 3:**
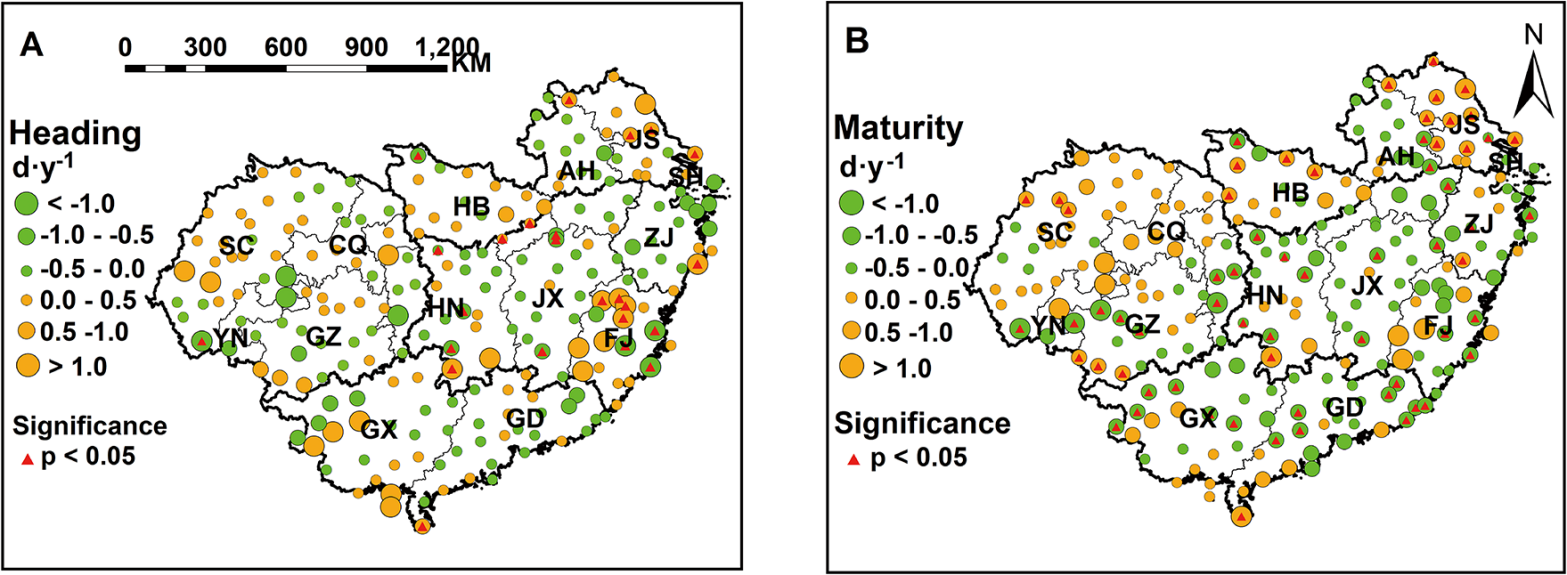
Annual trends of heading and maturity dates from 1981 to 2010 at each station. The size of the circle represents the magnitude of linear regression coefficients; Green and orange circles indicate negative and positive regression coeffiients, respectively; Red triangle indicates the significance of annual trend at this station (*p* < 0.05).

Rice phenological dates at 192 weather stations in South China from 1981 to 2010 were obtained from the Agrometeorological experimental stations (AES) operated by CMA and the provincial-level meteorological administrations. Phenological dates for the other 36 stations without an AES were replaced with the phenological dates of the best-fitted nearby AES [31]. Fig 2 showed the average values of the day of year (DOY) for heading and maturity dates during 1981–2010. Large differences of heading and maturity dates were observed at different stations. The maximum differences due to spatial variation in heading dates were 56 days and 35 days, and in maturity dates were 78 days and 34 days, for single-season rice and double-season early rice planting regions, respectively. The annual trends of heading and maturity dates during 1981–2010 at each station were showed in Fig 3. Negative trends were observed at 49.1% and 52.8% stations for heading and maturity dates, with 5.7% and 17.1% stations of significant (p < 0.05), respectively. Positive trends, almost as frequent as negative trends, were significant at 7.9% and 11.0% stations for heading and maturity dates, respectively. Overall, there were no consistent annual trends at all stations, and phenological dates were spatially and temporally varied among different stations. Therefore, the observed heading and maturity dates in each year from 1981 to 2010 were used at each station.

The observed datasets from AES also included the information of rice grain yield, cultivar characteristics and management practices at each station. Grain yield data from 1981 to 2010 were used for evaluating the effects of post-heading heat stress on rice grain yield. Local rice cultivars were planted and generally switched every 3–5 years. Crop management practices at each AES were generally same as or a little better than the local traditional practices.

### Data analysis

#### Description of heat stress indices

The threshold temperature of heat stress (T_h_) was differed among different cultivars, plant organs and developmental phases, and 35°C was used as T_h_ for the estimation of heat stress on rice grain yield in South China (S1 Fig). In our study, three heat stress indices were calculated with the historical daily maximum temperature (T_max_) dataset and the observed phenology dataset in each year at each weather station from 1981 to 2010. The calculation of heat stress indices was summarized in Table 1, in which the accumulated days of heat stress (ADHS) measured heat stress duration, and the heat stress intensity (HSI) measured the magnitude of heat stress. Moreover, an index named heat degree days (HDD) [31–33] was used to evaluate the comprehensive effects of duration and intensity of heat stress. It was calculated as follows:

$$HDD=\sum_{h}^{m}HD_{i}$$ (1)

$$HD_{i}=\{\begin{array}{c} 0\\ T_{\max_{i}}-{\text{T}}_{\text{h}} \end{array}\begin{array}{c} \;T_{\max_{i}}<{\text{T}}_{\text{h}}\\ \;T_{\max_{i}}\geq {\text{T}}_{\text{h}} \end{array}$$ (2)

where *h* and *m* are heading and maturity dates, respectively; T_maxi_ is the daily maximum temperature on day *i*; T_h_ indicates the threshold temperature of heat stress; HD_*i*_ indicates the heat degree days on day *i*, and HDD indicates the accumulated HD_*i*_ from heading to maturity.

**Table 1:** Definition of heat stress indices.

Index	Abbreviation	Unit	Definition
Accumulated days of heat stress	ADHS	d	Number of days when T_max_≥T_h_ from heading to maturity
Heat stress intensity	0030	°C	Average T_max_ in days when T_max_≥T_h_ from heading to maturity
Heat degree-days	HDD	°C·d	Accumulated heat degree days from heading to maturity, calculated as Eq (1) and Eq (2)

T_max_: daily maximum temperature; T_h_: the threshold temperature of heat stress.

#### Spatio-temporal variation of heat stress indices

All the heat stress indices were calculated annually at each station with actual heading and maturity dates obtained from AESs. The average value of each index at each station during 1981–2010 was determined to show the spatial variation of post-heading heat stress in the study region. The display of spatial characteristics for heat stress was analyzed in the software of ArcGIS 9.3 (Esri, Inc.), with the interpolation method of inverse distance weighting (IDW).

In order to detect the general temporal change from 1981 to 2010, annual trends of heat stress indices (Tr) at each station were calculated by fitting the time series of each index over 1981–2010 with linear regression.

#### Effects of phenology shift on trends of heat stress

Since heat stress indices were calculated with daily maximum temperature and the actual phenological dates at each station in each year, annual trends of heat stress indices (Tr) were therefore affected both by the change of maximum temperature, and by phenology shift during 1981–2010. In order to obtain a general response of heat stress to phenology shift, trends of heat stress indices only affected by the change of maximum temperature (Tr_mxt_) should be quantified and excluded. For this purpose, heat stress indices in each year were recalculated with the fixed phenological dates (i.e. the average heading and maturity dates from 1981 to 2010 as showed in Fig 2) at each station, and their trends were then fitted with linear regression. Thus, the difference between Tr and Tr_mxt_ generally represented the effects of phenology shift on annual trend of heat stress (Tr_phe_) from 1981 to 2010. Tr, Tr_mxt_ and Tr_phe_ were analyzed at each station of the sub-region. Tr and Tr_mxt_ of all the stations in each sub-region were compared by a paired t-test (p<0.05 and p<0.01) to determine whether their differences (i.e. Tr_phe_) were significant.

#### Effects of heat stress on rice grain yield

The year-to-year variation of climatic and grain yield data were both derived from the first-difference method which was commonly used to estimate the effects of climate change on crop grain yield [31, 34]. In order to exclude the variation of management and other non-climatic factors on rice grain yield variation, the first-difference yield was normalized with the average yield of previous three years (Eq 3) as proposed by Iizumi et al. [35]:

$$\Delta Y_{i}=\frac{Y_{i}\text{-}Y_{i\text{-}1}}{{\bar{Y}}_{i\text{-}3:i\text{-}1}}$$ (3)

where Δ*Y*_*i*_ is the normalized first-difference yield (ranges 0–1) in year *i*; *Y*_*i*_ and *Y*_*i*-1_ are the observed yield in year *i* and year *i*-1; ${\bar{Y}}_{i\text{-}3:i\text{-}1}$ indicates the average yield from year *i*-3 to year *i*-1, and the same ${\bar{Y}}_{i\text{-}3:i\text{-}1}$ was used in the first four years.

The index of growing degree days (GDD) was commonly used to estimate crop yield response to temperature variation in previous studies [36, 37], and it reflected the effective accumulated temperature during the crop growth season. However, the importance of heat stress effects was emphasized as well in recent years [10, 38, 39], because of the obvious increasing hot days during crop growth. Therefore, the statistical model for grain yield variation in response to temperature variation was preliminarily proposed as Eq (4):

$$\Delta Y_{i}=\beta_{0}+\beta_{1}\Delta GDD_{{}_{i}}+\beta_{2}\Delta HDD_{i}+\varepsilon$$ (4)

where GDD (°C·d) is calculated by the accumulation of daily average temperature above 10°C (the base temperature for rice growth) between heading and maturity [40]. ΔGDD_*i*_ and ΔHDD_*i*_ are the year-to-year variation of GDD and HDD (without normalization). β parameters are regression coefficients, and ε indicates the random error. β_1_ and β_2_ represent the sensitivities of grain yield to the variation of GDD and HDD, respectively, which are expressed as the percentage of grain yield changes since ΔY_*i*_ is the normalized year-to-year grain yield.

For the further determination of main temperature variables affecting yield variation, a partial correlation analysis was used to investigate the relationship between ΔY_*i*_ and the year-to-year variation of temperature variables (ΔGDD_*i*_ and ΔHDD_*i*_) among different sub-regions with all the stations. This method analyzed the correlation between ΔY_*i*_ and ΔHDD_*i*_ (or ΔGDD_*i*_) with the common variance between ΔHDD and ΔGDD removed. Temperature variables with the correlation coefficients of statistical significance (p<0.05) were considered as the main factors affecting yield variation in each sub-region. Corresponding temperature variables were eventually included in the statistical model to estimated yield variation.

The sensitivities of grain yield (β_1_ and β_2_) to temperature variables were investigated at each station of four sub-regions. Because of the limited observed samples for rice yield from 1981 to 2010 at each station, a bootstrap resampling method was used to estimate the uncertainty of samples [28, 37]. A total of 1000 bootstrap resamples was performed and the median value was used as the final regression coefficients of statistical model at each station. The sensitivities of grain yield at sub-region scale were estimated with the average values of all stations in each sub-region.

The total contribution of post-heading heat stress to yield variation (as a percentage) in rice from 1981 to 2010 at each sub-region was obtained by multiplying the sensitivity of grain yield to HDD (i.e. β_2_ in Eq (4)) with the variation of HDD during 1981–2010 at sub-region scale [28, 37]. The later was estimated by the linear trend of HDD from 1981 to 2010 (i.e. Tr).

Statistical processing was performed with SPSS 18.0 (SPSS Inc.) and R program (R Core Team, 2014). Significance was tested with two-tailed t-test at p<0.05 and p<0.01.

## Results

### Spatial variation of post-heading heat stress in South China

**Fig 4:**
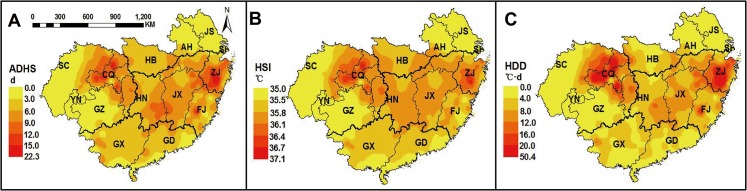
Spatial variation of post-heading heat stress indices from 1981 to 2010. Data above were calculated based on the observed phenological dates of each year at each station. ADHS, accumulated days of heat stress; HSI, heat stress intensity; HDD, heat degree days.

Distinct spatial variation of post-heading heat stress was showed in the study region from 1981 to 2010, based on the observed phenological date of each year at each station (Fig 4). The spatial variation for the average values during 1981–2010 of each heat stress index indicated that the post-heading heat stress was more serious in the central areas of the study region, including east S-SWP and west S-NMLYtz in single-season rice region and DE-SMLYtz in double-season rice region, than in the other areas. Moreover, larger spatial variation of heat stress was observed in the single-season rice region (S-SWP and S-NMLYtz), with obvious east-west spatial difference, than that in the double-season early rice region.

The average value of ADHS after heading during 1981–2010 in east SWP, west S-NMLYtz and DE-SMLYtz could be up to 10.4 days (d), 4.2 d more than that in the other parts of the study region. The average HSI after heading in the central areas of the study region was 36.4°C, 0.9°C higher than that in the other areas. The average HDD after heading in east S-SWP and DE-SMLYtz was about 14.5°C·d, three times higher than that in S-NMLYtz and DE-SC, and nearly six times the value in west S-SWP. The areas with the most serious heat stress in the single-season rice region and double-season early rice region were Chongqing (CQ) municipality in S-SWP and Zhejiang (ZJ) province in DE-SMLYtz, where the ADHS after heading were up to 11.2 d and 10.6 d, the HSI after heading were up to 36.5°C and 36.3°C, and the HDD after heading were up to 28.6°C·d and 24.3°C·d on average during 1981–2010 (Fig 4).

### Temporal trends of post-heading heat stress from 1981 to 2010

**Fig 5:**
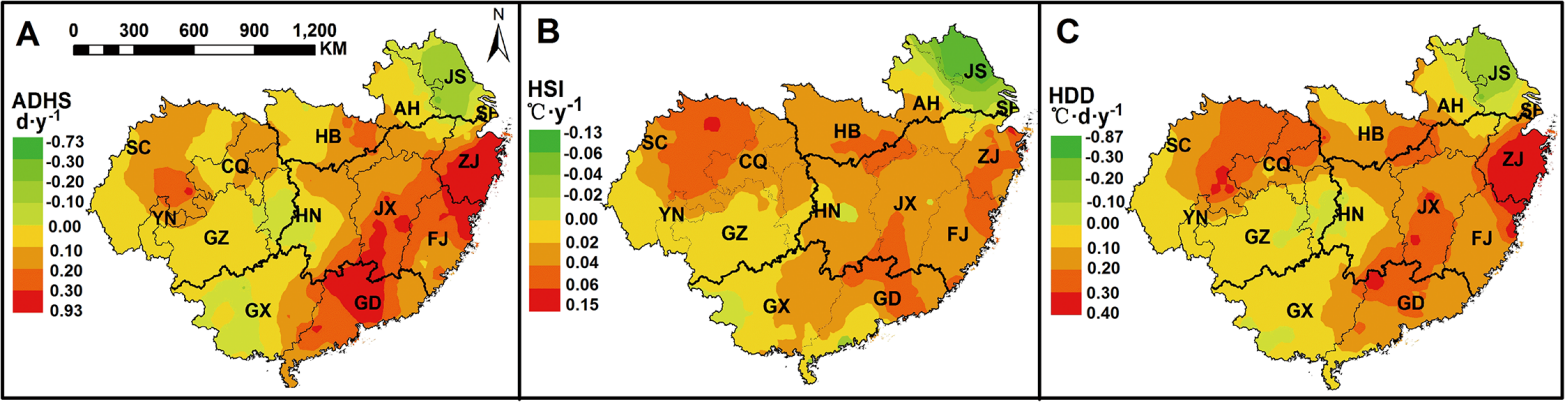
Temporal trends of post-heading heat stress indices from 1981 to 2010. Data above were calculated based on the observed phenological dates of each year at each station. ADHS, accumulated days of heat stress; HSI, heat stress intensity; HDD, heat degree days.

From 1981 to 2010, post-heading heat stress in rice increased in the study region except for some areas in the northeast (Fig 5). The post-heading heat stress decreased in east S-NMLYtz from 1981 to 2010, including Jiangsu (JS) province and most northern areas of Anhui (AH) province, and the average reduction for ADHS in east S-NMLYtz was about 0.06 d·y^-1^, and for HDD was 0.10°C·d·y^-1^.

Nevertheless, there was an obvious increase for post-heading heat stress in the east of double-season early rice region and in west S-SWP (most in northwest Sichuan province) of single-season rice region. This suggested that despite generally being a cooler area (annual average temperature during 1981–2010 was about 10.8°C), west S-SWP had an increasing occurrence for post-heading heat stress. ADHS showed an uptrend of 0.12 d·y^-1^ in the east of the double-season early rice region, and of 0.05 d·y^-1^ in west S-SWP. The increasing trends of HSI in the east of the double-season early rice region and in west S-SWP were 0.042°C·y^-1^ and 0.040°C·y^-1^, respectively. Trends for HDD were more than 0.2°C·d·y^-1^ both in west S-SWP and in the east of double-season early rice region.

### Effects of phenology shift on temporal trends of heat stress indices during 1981–2010

**Fig 6:**
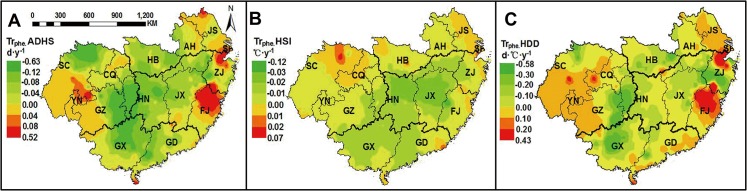
Effects of phenology shift on temporal trends of heat stress indices from 1981 to 2010. ADHS, accumulated days of heat stress; HSI, heat stress intensity; HDD, heat degree days.

Phenology shift affected the temporal trends of post-heading heat stress obviously in the whole study region from 1981 to 2010, with generally similar trends among ADHS, HSI and HDD (Fig 6). Combined with the findings from Figs 5 and 6, phenology shift from 1981 to 2010 mitigated the increasing trends of post-heading heat stress in most central areas of South China (at 83.2% stations), while accelerated the increasing trends of post-heading heat stress in west S-SWP and Fujian (FJ) province of southeast DE-SMLYtz. In particular, the decreasing trend of post-heading heat stress was observed in east S-NMLYtz (mostly in Jiangsu (JS) province) (Fig 5), and phenology shift during 1981–2010 continually accelerated this decreasing trend (Fig 6).

Because HDD was the comprehensive index that evaluated both heat stress duration and intensity, Table 2 analyzed the effects of phenology shift on temporal trends of post-heading heat stress using HDD index at different sub-region scales. Owing to phenology shift, the increasing trends of post-heading heat stress decreased significantly in S-NMLYtz (from 0.042°C·d·y^-1^ to 0.031°C·d·y^-1^), DE-SMLYtz (from 0.590°C·d·y^-1^ to 0.323°C·d·y^-1^) and DE-SC (from 0.249°C·d·y^-1^ to 0.178°C·d·y^-1^), while did not mitigate in S-SWP (p = 0.842). Overall, the effects of phenology shift on the temporal trend of post-heading heat stress were significant in the whole study region, with higher significant level in double-season early rice region.

**Table 2:** The paired t-test on temporal trends of heat degree days (HDD) driven by different influencing factors in each sub-region.

Regions	Average trends of HDD driven by phenology shift and by variations of maximum temperature Tr.ave (°C·d·y^-1^)	Average trends of HDD driven by variations of maximum temperature Tr_mxt_.ave (°C·d·y^-1^)	Average trends of HDD driven by phenology shift Tr_phe._ave (°C·d·y^-1^)	P-value
S-NMLYtz	0.031	0.042	-0.011	0.000**
S-SWP	0.245	0.110	0.135	0.842
DE-SMLYtz	0.323	0.590	-0.267	0.046*
DE-SC	0.178	0.249	-0.071	0.000**
SR	0.006	0.102	-0.096	0.379
DER	0.276	0.455	-0.179	0.010**
South China	0.175	0.311	-0.136	0.018*

Tr.ave, Tr_mxt_.ave and Tr_phe_.ave are the average value of regression coefficients at all stations of each sub-region. S-NMLYtz, single-season rice sub-region in the northern Middle and Lower Reaches of Yangtze River; S-SWP, single-season rice sub-region in Southwest Plateau; DE-SMLYtz, double-season early rice sub-region in the southern Middle and Lower Reaches of Yangtze River; DE-SC, double-season early rice sub-region in Southern China. SR, single-season rice region; DER, double-season early rice region.

*, significant at p < 0.05

**, significant at p < 0.01.

### The impact of heat stress on rice grain yield

#### Correlation between rice grain yield and temperature variables

Table 3 was the partial correlation analysis on the relationship of ΔHDD and ΔGDD with ΔY. Significant negative correlations were observed between ΔY and ΔHDD among four sub-regions in South China, suggesting obvious grain yield loss due to the increase of post-heading heat stress from 1981 to 2010. The variation of post-heading GDD only affected yield variation in S-SWP, and the significance level of the correlation between ΔY and ΔGDD (p = 0.029) was less than that between ΔY and ΔHDD (p = 0.000). These results indicated that post-heading heat stress was more important for rice grain yield variation among the four sub-regions during 1981–2010, as compared with effective accumulated temperature.

**Table 3:** Partial correlation analysis on the relationship of ΔHDD and ΔGDD with ΔY in each sub-region of South China.

Sub-region	Temperature variable	Partial correlation coefficient	P value
0030	ΔHDD	-0.219	0.026*
	ΔGDD	0.089	0.415
S-SWP	ΔHDD	-0.296	0.000**
	ΔGDD	0.124	0.029*
DE-SMLYtz	ΔHDD	-0.275	0.023**
	ΔGDD	-0.106	0.120
DE-SC	ΔHDD	-0.453	0.000**
	ΔGDD	-0.028	0.481

S-NMLYtz, single-season rice sub-region in the northern Middle and Lower Reaches of Yangtze River; S-SWP, single-season rice sub-region in Southwest Plateau; DE-SMLYtz, double-season early rice sub-region in the southern Middle and Lower Reaches of Yangtze River; DE-SC, double-season early rice sub-region in Southern China.

*, significant at p < 0.05

**, significant at p < 0.01.

#### Sensitivity of grain yield to the increasing temperature

**Fig 7:**
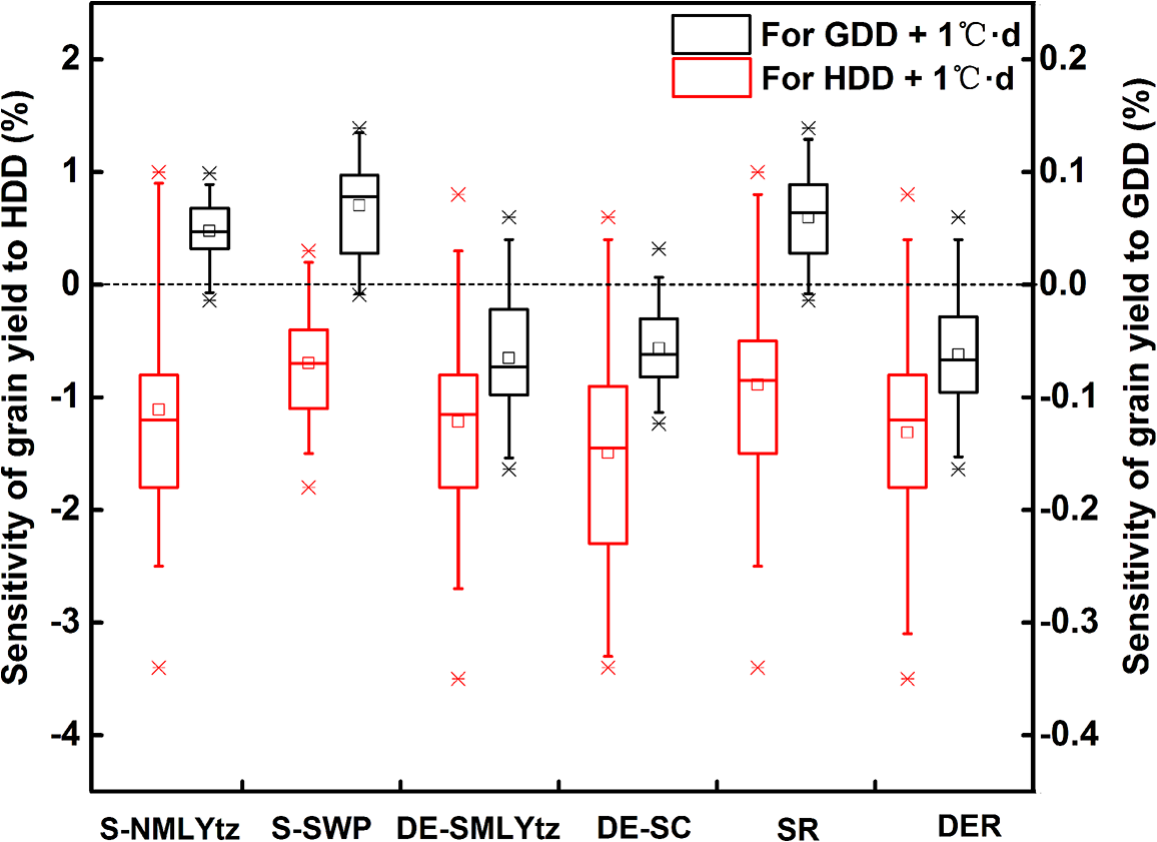
Boxplots of the sensitivities of grain yield to post-heading HDD and GDD in different sub-regions. Sensitivities of grain yield were estimated due to the increase of per-unit HDD and per-unit GDD during post-heading stage in rice at different stations of each sub-region. The upper and lower boundaries of the box indicate the 75th and 25th percentiles, respectively. The above and below whiskers indicate the 95th and 5th percentiles, respectively. The line within the box marks the median value and the pane in the box indicates the mean value. S-NMLYtz, single-season rice sub-region in the northern Middle and Lower Reaches of Yangtze River; S-SWP, single-season rice sub-region in Southwest Plateau; DE-SMLYtz, double-season early rice sub-region in the southern Middle and Lower Reaches of Yangtze River; DE-SC, double-season early rice sub-region in Southern China. SR, single-season rice region; DER, double-season early rice region.

Yield variation due to the increasing temperature was estimated both by considering GDD and HDD (type Ⅰ)_,_ and by considering only HDD (type Ⅱ) during the post-heading stage. Table 4 showed that there was no obvious improvement for determination coefficients (R^2^) of the statistical model of type Ⅰ in S-NMLYtz, DE-SMLYtz and DE-SC, as compared with that of type Ⅱ, suggesting yield variation in these sub-regions was mainly affected by post-heading heat stress. However, yield variation in S-SWP was affected both by heat stress and by effective accumulated temperature during post-heading stage, as indicated by the change of R^2^ between the two types of statistical models. These results were consistent with the partial correlation analysis between rice grain yield and temperature variables (Table 3). With the selected statistical model at different sub-regions, the sensitivities of grain yield to post-heading HDD (yield change for each 1°C·d increase of HDD) in rice were -1.2%, -0.9% and -1.1% in S-NMLYtz, DE-SMLYtz and DE-SC, respectively, as indicated by β_2_. In S-SWP, the sensitivities of grain yield were -0.8% and 0.09% with each 1°C·d increase of HDD and GDD during post-heading stage, respectively. Fig 7 showed that there were differences in the sensitivity of grain yield to temperature variables among different stations of each sub-region. Although there were some uncertainties at different stations, post-heading heat stress generally decreased rice grain yield in each sub-region and in the two planting systems (single-season rice and double season early rice) of South China.

**Table 4:** Regression coefficients and determination coefficients (R^2^) of statistical model for grain yield in response to temperature variables (per unit).

Sub-regions	Considering HDD and GDD (typeⅠ)				Considering only HDD (typeⅡ)		
	β_0_	β_1_	β_2_	R^2^	β_0_	β_2_	R^2^
S-NMLYtz	0.011	0.0005	-0.011**	0.138	**0.013**	-0.012**	**0.134**
S-SWP	**0.024**	0.0009*	-0.008**	**0.173**	0.021	-0.007**	0.112
DE-SMLYtz	0.005	-0.0007	-0.009**	0.215	**0.004**	-0.009**	**0.208**
DE-SC	0.007	-0.0006	-0.010**	0.295	**0.006**	-0.011**	**0.290**

β_1_ and β_2_ are the coefficients for ΔGDD and ΔHDD in Eq (4), respectively, which represent the sensitivities of grain yield to temperature variables. Significance of β_1_, β_2_ was tested at p<0.05 (*) and p<0.01 (**). Numbers with black bold indicate the regression coefficients for the selected type of statistical model. S-NMLYtz, single-season rice sub-region in the northern Middle and Lower Reaches of Yangtze River; S-SWP, single-season rice sub-region in Southwest Plateau; DE-SMLYtz, double-season early rice sub-region in the southern Middle and Lower Reaches of Yangtze River; DE-SC, double-season early rice sub-region in Southern China.

#### Contribution of post-heading heat stress to yield variation from 1981 to 2010

**Fig 8:**
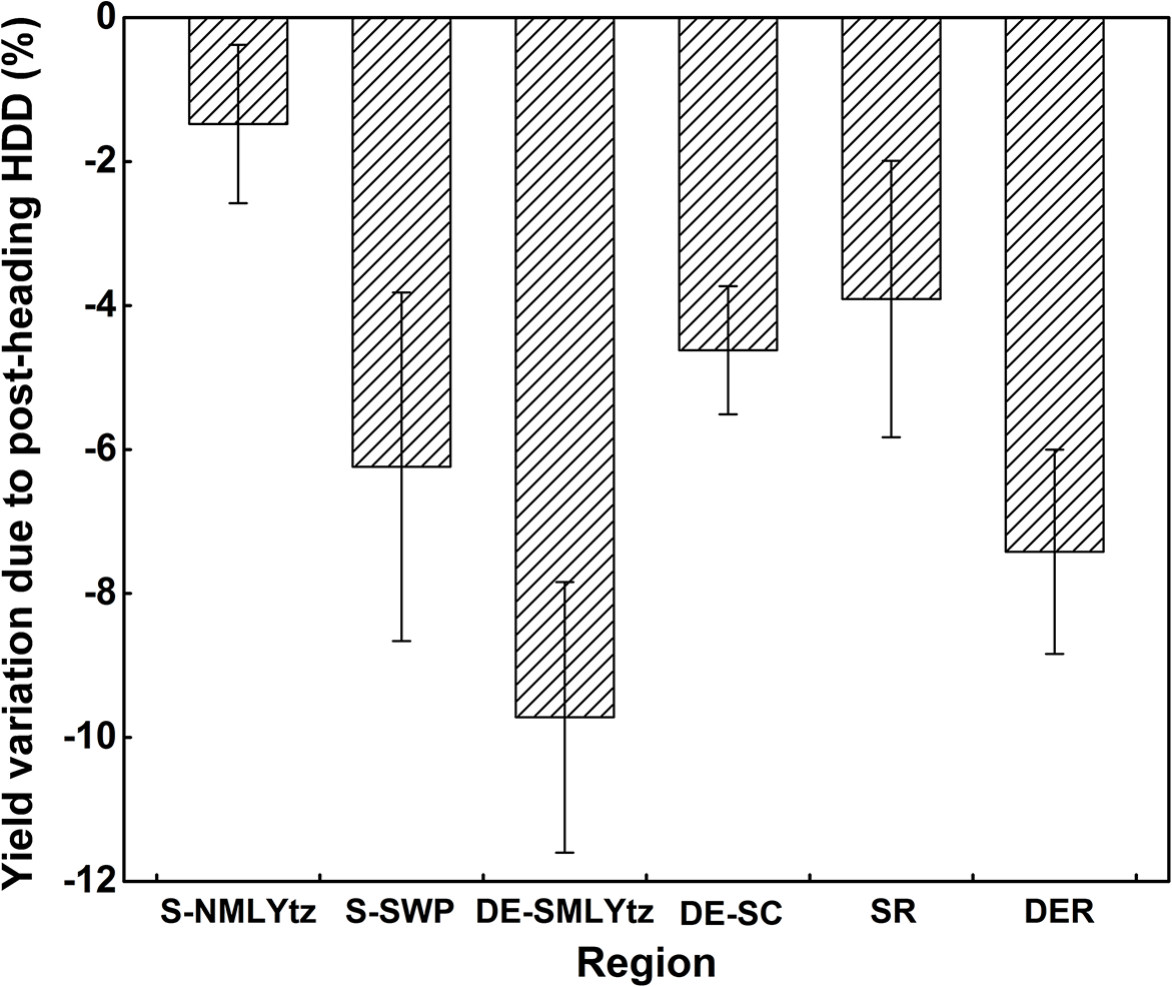
Contribution of post-heading heat stress to yield variation (%) during 1981–2010 in different sub-regions. Whiskers show the 95% confidence interval. S-NMLYtz, single-season rice sub-region in the northern Middle and Lower Reaches of Yangtze River; S-SWP, single-season rice sub-region in Southwest Plateau; DE-SMLYtz, double-season early rice sub-region in the southern Middle and Lower Reaches of Yangtze River; DE-SC, double-season early rice sub-region in Southern China. SR, single-season rice region; DER, double-season early rice region.

The total contribution of post-heading heat stress to rice yield variation at each sub-region from 1981 to 2010 was showed in Fig 8. From 1981 to 2010, post-heading heat stress generally decreased rice grain yield by 1.5%, 6.2%, 9.7% and 4.6% in S-NMLYtz, S-SWP, DE-SMLYtz and DE-SC, respectively. Rice production in double-season early rice region (S-NMLYtz and S-SWP) was more affected by post-heading heat stress due to climate warming than that in single-season rice region (DE-SMLYtz and DE-SC). Total yield losses from 1981 to 2010 were 3.9% and 7.4% with the increase of post-heading heat stress in single-season rice region and double-season early rice region, respectively.

## Discussion

### Effect of phenology shift on the trend of heat stress

Climate change significantly affected agricultural production in recent years [41, 42]. Phenology shift was recognized as the most direct response to climate warming [43, 44]. The increasing temperature during the past 2–3 decades are expected to accelerate phenological development in rice [27, 45], but cultivar improvement by breeding efforts have partially offset the phenology acceleration at most sites in China [28, 40, 46]. Temporal trends in phenological dates at all stations of South China confirmed above conclusion, since phenology acceleration was only observed at about half of the stations (Fig 3). However, previous studies seldom estimated the contribution of phenology shift to the trend of heat stress during the past few decades. Taking the spatio-temporal variation of phenological dates into consideration, our study indicated that phenology shift in rice mitigated the increasing trends of heat stress in the central areas of South China (Fig 6), where severe post-heading heat stress commonly occurred from 1981 to 2010 (Figs 4 and 5). This interesting finding suggested that breeding efforts (or cultivar improvement) during 1981–2010 efficiently reduced the risk of post-heading heat stress in rice in most areas of South China. In addition, phenology shift mitigated the increasing trends of post-heading heat stress much more in the two double-season rice sub-regions, while less or not in the two single-season rice sub-regions (Table 2). This was probably attributed to the less effects of heat stress on rice productions in two single-season rice sub-regions, and thus breeding target was not focused on, especially in west S-SWP (Fig 4).

Although phenology shift mitigated the uptrend of heat stress at temporal scale, the spatial variation of phenological dates directly affected the distribution and magnitude of post-heading heat stress among different regions. For example, severe post-heading heat stress was observed in east S-SWP and west S-NMLYtz, because the period from heading to maturity was about from early August to early September in east S-SWP, and from mid-July to mid-August in west S-NMLYtz, respectively, when were the hotter part of the year (Fig 2). The long exposure of high temperature during post-heading reproductive phase could cause severe heat stress in rice. Therefore, considering the spatio-temporal variation of phenological dates is important for the analysis of the magnitude of heat stress, otherwise different conclusions might be drawn.

### Spatial and temporal variation in post-heading heat stress

South China spans about 24° longitude and 15° latitude with a warm and humid climate in rice growing season. Rice planted in South China is subject to various types of climate, and thus the spatial variation of heat stress is obvious over the whole rice production region. During 1981–2010, post-heading heat stress was more serious in the central areas of South China where double-season early rice widely planted. The spatial variations of post-heading heat stress generally reflected the differences of each sub-region in response to climate warming. Phenology variation is one of the main factors affecting the spatial differences of heat stress among sub-regions. The severe heat stress in DE-SMLYtz and east S-SWP was mainly due to the exposure of reproductive phase after heading to the hotter part of summer season. Similarly, more spatial variation of post-heading heat stress was observed in single-season rice region because of the greater variation of heading and maturity dates, than that in double-season early rice region (Fig 2).

With or without phenology shift, temporal trends of post-heading heat stress in rice both increased in most areas of South China from 1981 to 2010, especially in west S-SWP and in the east of double-season early rice region (Fig 5 and Table 2). These results indicated that post-heading heat stress would become more severe if the trends persisted in the future. In particular, more attentions should be paid to the sub-region in SWP, where a high level of heat stress in the east (Fig 4) and an increasing trend of heat stress in the west were observed (Fig 5). Therefore, rice planted in S-SWP in the near future might suffered the potential threaten of post-heading heat stress. Although the trend of post-heading average temperature changed little from 1981 to 2010 for rice in DE-SC (S1 Table), the warmer area in South China, post-heading heat stress still increased significantly (Table 3). This suggested that post-heading heat stress would be a considerable increase with climate warming in the general warm areas of South China in the future.

### Quantification of the effect of temperature variables on grain yield

Climatic warming in the last two or three decades has had great impacts on crop production across the world [34, 40, 47]. Previous studies paid more attentions to the effects of average temperature of growing season on grain yield [28, 42]. However, little was known about the influence of heat stress on crop production. With the increasing variation of temperature under climate warming, the effect of hot days during crop growth was emphasized recently [28, 38], and some studies even considered it as the main variable determining grain yield in statistical models [10]. In our study, possible impacts of effective accumulated temperature and heat stress on rice grain yield were preliminarily considered in Eq (4), and model selection was then performed by analyzing the significance of the correlation between yield variable and temperature variables (Tables 3 and 4). Generally, HDD was more appropriate than ADHS and HSI to estimate yield variation together with GDD during post-heading stage of rice in South China, as the R^2^ of the regression equations indicated (Table 4, S2 Table and S3 Table). Moreover, no matter which heat stress indices were used, significant correlations between heat stress indices and yield variation were found in our study. These results demonstrated the relative importance of post-heading heat stress for rice grain yield variation in South China during 1981–2010.

With a warming climate in South China, temperature during post-heading stage was considered as the main climatic factor affecting rice yield in this study. Other factors such as CO_2_, solar radiation, precipitation might also play vital roles on grain yield in field conditions [31, 47]. Empirical equation for yield estimation with multiple variables or considering time-varying effects of equation parameters would achieve high determination coefficients. However, exploring the most appropriate statistical equation for yield estimation was beyond the focus of this study, and our study emphasized the dominated role that heat stress played on rice yield in South China since 1980s. Till now, the accurate quantification of the independent effect of heat stress on grain yield from other variables (e.g. average temperature, maximum temperature, precipitation) is difficult with statistical model because of the complex effects among them. As an alternative, the process-based crop model makes it possible to isolate the effects of different climate factors on grain yield formation [38]. However, the poor predictions and high systematic errors of process-based crop models under heat stress were reported in many studies [20, 33, 48]. Recently, the process-based sub-model for phenology simulation under heat stress was improved [48], while more validations are still needed with the field experiment dataset under heat stress [49]. Future study will pay more attention to evaluate the impact of heat stress on grain yield with the improved process-based crop models.

### Uncertainties in the effects of heat stress on rice grain yield

An uncertainty for the effects of heat stress on rice grain yield might be from the selection of high temperature threshold. The actual value of high temperature threshold during post-heading reproductive phase is impossible to be determined at different stations for various rice cultivars. In order to select the optimal high temperature threshold in the whole study region, the method of leave-one-out cross validation (LOOCV) was used to find the minimized mean square errors for grain yield predictions among different temperature thresholds (range from 30°C to 38°C with an interval of 0.2°C) (S1 Fig). Results showed that the 35°C was the optimal high temperature threshold in rice in South China for the two types of statistical models. In addition, since there was great uncertainty in yield variation at a single station from 1981 to 2010 (Fig 7), the effects of post-heading heat stress on rice grain yield was eventually estimated at sub-region scale (Fig 8). Generally, our results suggest that the historical increase of post-heading heat stress has resulted in the reduction of rice grain yield in most rice cultivation regions of South China from 1981 to 2010, averagely 3.9% and 7.4% in the single-season rice region and double-season early rice region, respectively (Fig 8). With these percentages, rice production in South China during 1981–2010 reduced 6.73×10^7^t and 8.89×10^7^t in single-season rice region and double-season early rice region because of historical post-heading heat stress, respectively, according to the statistical planting area at provincial level (National Bureau of Statistics of China).

### Targeted adaptation strategies

Recent studies have emphasized that more attentions should be paid to the increasing temperature variation under heat stress [39, 50]. Our results showed that there was clear spatio-temporal variation in post-heading heat stress during rice growing season across South China. Consequently, the specific adaptation or mitigation strategies should be suggested for each sub-region. Serious post-heading heat stress during 1981–2010 was observed in west S-NMLYtz and east S-SWP of single-season rice region, where rice growth between heading and maturity was exposed to the highest temperature environment in a year (in mid-July and mid-August) (Fig 2A and 2B). Therefore, besides the improvement of cultivar heat resistance, the adjustment of sowing date in rice would be very important to avoid the occurrence of post-heading heat stress. Furthermore, although there was less heat stress in west-SWP (mostly in Sichuan, SC), great increasing trend of heat stress was observed from 1981 to 2010 (Figs 4 and 5). With a large sown area (Fig 1A), SC provides the largest production of single-season rice in China. Nevertheless, there was no superiority for the agricultural practice (e.g. irrigation areas) in SC (S2 Fig), and phenology shift from autonomous breeding did not mitigate the increasing trend of post-heading heat stress in most areas of SC (Fig 6). Therefore, rice yield per unit enhances less in SC since 1980s as indicated by the observations at AES stations (S2 Fig) and previous study [26]. These results suggest that the progress of agricultural practice (e.g. irrigation) changes little in most areas of SC from 1981 to 2010. In contrast, though little heat stress in Jiangsu (JS) (the second producer of single-season rice), the progress of agricultural practice contributes a lot to rice production [51]. Hence, the improvement of management practices to mitigate the effects of heat stress will benefit the stabilization of rice yield in SC in the future. During 1981–2010, double-season early rice region experienced severe post-heading heat stress in most areas, such as the two largest producers, Hunan (HN) and Jiangxi (JX) provinces (Fig 1A). With crop rotation of early rice and late rice, the light and temperature resources can be efficiently used in double-season rice planting region. Though cultivar changes with the phenology shift partly decreases the uptrend of heat stress (Fig 6 and Table 2), more efforts on breeding are still emphasized. Cultivars with heat-resistance or heat-escape traits are suggested to be widely planted in double-season early rice region, because heat stress might persist in most areas according to current uptrends (Fig 5).

## Conclusion

Large spatial and temporal variations of post-heading heat stress in rice were observed in South China, with differences among four sub-regions. The spatial variation of heat stress was greater in the single-season rice region than the double-season early rice region. The most serious heat stress occurred in the central areas of South China according to the average values of heat stress indices from 1981 to 2010. Post-heading heat stress increased in most rice planting regions of South China during 1981–2010, with the higher uptrends in the east of double-season early rice region and west Sichuan (SC) province. Phenology shift mitigated the increasing trend of post-heading heat stress in most central areas of South China during 1981–2010. Post-heading heat stress played the dominated role on rice yield variation among four sub-regions in South China, as compared with the effective accumulated temperature during post-heading growth season. Despite some uncertainties, post-heading heat stress averagely decreased rice production by 3.9% and 7.4% in single-season rice region and double-season early rice region, respectively. Rice production across South China was affected by the post-heading heat stress since 1980s, and the specific adaptation or mitigation strategies are needed for different sub-regions to ensure food security under climate change.

## Supporting Information

S1 Fig Leave-one-out cross validation (LOOCV) for the determination of high temperature threshold by fitting two types of statistical model for yield estimation. (DOCX)

S2 Fig Rice grain yield per unit area (A) and irrigation area (B) in Sichuan (SC) and Jiangsu (JS) provinces from 1981 to 2010. (DOCX)

S1 Table Annual average temperature (AT_avg_) and trends of post-heading average temperature (Tr_at_) from 1981 to 2010 among four sub-regions in South China. (DOCX)

S2 Table Regression coefficients and determination coefficients (R^2^) of statistical model for grain yield in response to ADHS and GDD. (DOCX)

S3 Table Regression coefficients and determination coefficients (R^2^) of statistical model for grain yield in response to HSI and GDD. (DOCX)
