# Supplementary material for: Post-Heading Heat Stress in Rice of South China during 1981-2010
Source: PLoS One. 2015 Jun 25;10(6):e0130642. doi: 10.1371/journal.pone.0130642 (PMC4482448; doi:10.1371/journal.pone.0130642)
Supplement: S1 Fig — (DOCX) [file pone.0130642.s001.docx]

**S1 Fig. Leave-one-out cross validation (LOOCV) for the determination of high temperature threshold by fitting two types of statistical model for yield estimation.** LOOCV is a validation method to estimate model performance on the out-of-sample, which usually removes one-year data in the series, fits the model using remaining data and predicts the removed years. Heat degree days (HDD) are calculated with different high temperature threshold (range from 30℃ to 38℃with an interval of 0.2℃), and model parameters are re-estimated. Then LOOCV is used to test the model performance with cv.glm() function of "boot" package in R program. The optimal high temperature threshold is eventually determined according to the lowest root-mean-square error (RMSE) of the statistical models. Here two types of statistical models that consider only HDD and the interaction of HDD and GDD are both estimated.
