# Supplementary material for: Post-Heading Heat Stress in Rice of South China during 1981-2010
Source: PLoS One. 2015 Jun 25;10(6):e0130642. doi: 10.1371/journal.pone.0130642 (PMC4482448; doi:10.1371/journal.pone.0130642)
Supplement: S2 Fig — (DOCX) [file pone.0130642.s002.docx]

**

**

**S2 Fig. Rice grain yield per unit area (A) and irrigation area (B) in Sichuan (SC) and Jiangsu (JS) provinces from 1981 to 2010.** Data of rice yield per unit area in each year were the average value of all the Agrometeorological experimental stations in SC and JS; Data of irritation area were from National Bureau of Statistics of China.
