# Supplementary material for: Post-Heading Heat Stress in Rice of South China during 1981-2010
Source: PLoS One. 2015 Jun 25;10(6):e0130642. doi: 10.1371/journal.pone.0130642 (PMC4482448; doi:10.1371/journal.pone.0130642)
Supplement: S1 Table — (DOCX) [file pone.0130642.s003.docx]

**S1 Table. Annual average temperature (AT_avg_) and annual trends of post-heading average temperature (Tr_at_) from 1981 to 2010 among four sub-regions in South China.**

| Temperature variable | S-NMLYtz | S-SWP | DE-SMLYtz | DE-SC |
| --- | --- | --- | --- | --- |
| AT_avg_ (℃) | 12.1 | 15.3 | 17.6 | 21.2 |
| Tr_at_ (℃·y^-1^) | 0.02^*^ | 0.03^*^ | 0.05^*^ | 0.01 |

Both of the two temperature variables above were obtained from the average value of all the stations in each sub-region. Statistical significance for Tr_at_ was tested at p < 0.05 (^*^) and p < 0.01 (^**^). S-NMLYtz, single-season rice sub-region in the northern Middle and Lower Reaches of Yangtze River; S-SWP, single-season rice sub-region in Southwest Plateau; DE-SMLYtz, double-season early rice sub-region in the southern Middle and Lower Reaches of Yangtze River; DE-SC, double-season early rice sub-region in Southern China.
