# Supplementary material for: Post-Heading Heat Stress in Rice of South China during 1981-2010
Source: PLoS One. 2015 Jun 25;10(6):e0130642. doi: 10.1371/journal.pone.0130642 (PMC4482448; doi:10.1371/journal.pone.0130642)
Supplement: S2 Table — (DOCX) [file pone.0130642.s004.docx]

**S2 Table. Regression coefficients and determination coefficients (R^2^) of statistical model for grain yield in response to ADHS and GDD.**

| Sub-region | Considering ADHS and GDD | | | |  | Considering only ADHS | | |
| --- | --- | --- | --- | --- | --- | --- | --- | --- |
|  | β_0_ | β_1_ | β_2_ | R^2^ |  | β_0_ | β_2_ | R^2^ |
| S-NMLYtz | 0.015 | 0.0005 | -0.014^**^ | 0.117 |  | 0.015 | -0.014^**^ | 0.113 |
| S-SWP | 0.029 | 0.0009^*^ | -0.012^**^ | 0.149 |  | 0.023 | -0.013^**^ | 0.105 |
| DE-SMLYtz | 0.012 | -0.0007 | -0.015^**^ | 0.172 |  | 0.008 | -0.015^**^ | 0.168 |
| DE-SC | 0.011 | -0.0006 | -0.017^**^ | 0.223 |  | 0.010 | -0.016^**^ | 0.217 |

Statistical models used above were ∆Y*_i_* =*β*_0_+*β*_1_∆GDD*_i_* +*β*_2_∆ADHS*_i_* +ε and ∆Y*_i_* =*β*_0_+*β*_2_∆ADHS*_i_* +ε, respectively. Significance of β_1_, β_2_ was tested at p<0.05 (^*^) and p<0.01 (^**^). S-NMLYtz, single-season rice sub-region in the northern Middle and Lower Reaches of Yangtze River; S-SWP, single-season rice sub-region in Southwest Plateau; DE-SMLYtz, double-season early rice sub-region in the southern Middle and Lower Reaches of Yangtze River; DE-SC, double-season early rice sub-region in Southern China.
